# The ability of GLIM and MNA-FF to diagnose malnutrition and predict sarcopenia and frailty in hospitalized adults over 60 years of age

**DOI:** 10.3389/fnut.2024.1456091

**Published:** 2024-11-08

**Authors:** Gabriella D. da Silva, Afra V. De A. Batista, Maria C. R. De A. Costa, Ana C. O. dos Santos

**Affiliations:** ^1^Institute of Biological Sciences, University of Pernambuco, Recife, Brazil; ^2^Oswaldo Cruz University Hospital, University of Pernambuco, Recife, Brazil; ^3^Faculty of Medical Sciences, University of Pernambuco, Recife, Brazil

**Keywords:** calf circumference, fat-free mass index, GLIM, muscle mass, nutritional assessment, nutritional screening

## Abstract

**Introduction:**

Malnutrition remains common in adults over 60 years old. Although there are screening and diagnostic tools for malnutrition, there is no globally used approach to diagnosing malnutrition in older adults admitted to the hospital. In this study, we verified the agreement between the Global Leadership Initiative on Malnutrition (GLIM) and the Mini Nutritional Assessment (MNA) and the ability of the instruments to identify frailty and sarcopenia associated with malnutrition.

**Methods:**

For adults over 60 years old, malnutrition diagnosis was performed using the Mini Nutritional Assessment Full Form (MNA-FF) tool and the GLIM criteria, which included calf circumference and fat-free mass index to assess muscle mass, with and without the Mini Nutritional Assessment Short Form (MNA-SF) screening. Health conditions were assessed in older adults, and the association of these conditions with malnutrition was analyzed using both tools.

**Results:**

A total of 432 adults over 60 years old were investigated with a mean age of 71.14 ± 8 years. The GLIM criteria with the nutritional screening tool identified 61–63% of older adults as malnourished. Of these, 63–64% were severely malnourished. The MNA-FF tool classified 20% of those assessed as malnourished. The agreement between the MNA-FF and GLIM was better with the use of screening, with a kappa (K) value of −0.10 and − 0.11. Sarcopenia was associated with malnutrition as identified by the MNA-FF (OR: 3.08, 95% CI: 1.84–5.14) and only by the GLIM ANTHRO (OR: 1.66, 95% CI: 1.05–2.63). Frailty was associated with the MNA-FF (OR: 15.99, 95% CI: 2.16–118.36), GLIM ANTHRO (OR: 2.21, 95% CI: 1.31–3.71), and GLIM BIA (OR: 2.45, 95% CI: 1.45–4.12).

**Conclusion:**

It is possible to verify that divergent conceptual frameworks are used to understand malnutrition by the MNA-FF and GLIM and that the GLIM obtained a greater number of malnutrition diagnoses. Both the GLIM ANTHRO and the MNA-FF associated malnutrition with frailty and sarcopenia, with higher hazard ratios for the MNA-FF.

## 1 Introduction

Malnutrition remains common in older populations around the world. Current estimates indicate that around a quarter of older adults (65 years and older) are malnourished or at risk of malnutrition (1). This number is likely to increase along with the rapid increase in the aging population. The United Nations (UN) predicts that, between 2019 and 2050, the population aged 65 and older will double in many regions (2). To reduce malnutrition in older populations, a timely and concerted effort is needed to prioritize, prevent, recognize, and adequately manage malnutrition in this age group (3).

There are no simple and unequivocal criteria to diagnose malnutrition mainly those associated with nutritional and clinical conditions with high specificity and sensitivity (4). The Mini Nutritional Assessment (MNA) is considered one of the most validated tools for identifying malnutrition or risk of malnutrition in older adults (5, 6). However, the Mini Nutritional Assessment Full Form (MNA-FF) has disadvantages, including subjective questions that are not appropriate for older adults who are hospitalized, difficulty in administering it to older adults with cognitive impairment, and a completion time of 10 to 15 min (7). Even so, it is validated as a good nutritional screening tool (8). Thus, various tools have been applied to quickly identify malnutrition in hospitalized older adults, each with its own strengths and weaknesses (7).

The Global Leadership Initiative on Malnutrition (GLIM) published a proposal to facilitate malnutrition diagnoses with more specific and objective criteria in an approach that begins with nutritional risk screening followed by two steps, one of which consists of a more in-depth assessment to diagnose malnutrition. GLIM consists of three phenotypic criteria (reduced body mass index (BMI), unintentional weight loss, and reduced muscle mass) and two etiological criteria (reduced dietary intake/impaired nutrient assimilation and inflammation or disease burden). The presence of at least one of each type of criterion contributes to establishing a malnutrition diagnosis, and phenotypic criteria can be used to classify severity (4).

Clinical practice will benefit from a validated nutritional assessment instrument in adults over 60 years of age admitted to the hospital. Thus, this research aimed to verify the malnutrition diagnosis agreement using the GLIM criteria compared to the MNA-FF in adults over 60 years of age admitted to the hospital, checking which of the two tools is best associated with the clinical conditions presented by adults.

## 2 Materials and methods

### 2.1 Participants

Participants in this study, aged 60 years or older, were recruited from a university hospital and admitted to the geriatric or medical clinic. They were included based on specific criteria: both genders, age 60 or over, with appropriate physical and clinical conditions as per the data collected from their medical records, and the ability to answer the required questions. Exclusion criteria included: bedridden individuals, which made anthropometric assessment (weight and height) impossible; the presence of edema in the extremities, anasarca, or ascites that could lead to weight overestimation; limb amputations; individuals ineligible for disease-modifying therapies based on their medical records; those with spinal cord injuries or compression; individuals with pacemakers, heart valves, or metal prostheses; corticotherapy treatment; individuals with severe liver or kidney disease; and individuals with consumptive syndromes. This study was conducted between June 2021 and June 2023. The surveys were carried out by the research team duly trained within 48 h after hospital admission and other information came from hospital records. The study was carried out in accordance with the ethical guidelines for human research, and informed consent was obtained from all participants. The study was approved by the Ethics Committee (REC Protocol Approval 4.949.371/September/2021) and steered under the Declaration of Helsinki.

### 2.2 Anthropometry—ANTHRO

Body weight and height were measured or estimated (9, 10), and arm circumference (AC), calf circumference (CC) (11), and body mass index (BMI) were calculated. For handgrip strength, a hydraulic hand dynamometer was used, and the maximum value of three measurements of the dominant hand was used (12).

### 2.3 Electrical bioimpedance and body composition

Bioelectrical impedance analysis (BIA) was carried out using Sanny® equipment, specifically the tetrapolar BioSanny4 1,010 model. All measurements were performed under standardized conditions in accordance with the manufacturer’s protocol. The calculations were made using regression equations (13, 14) incorporated into the BIA software. Fat mass (FM), skeletal muscle mass (SMM), and fat-free mass (FFM) were assessed, with FFM being the final measure used to calculate the fat-free mass index (FFMI).

### 2.4 GLIM approach

In step 0 of the GLIM approach, the Mini Nutritional Assessment Short Form (MNA-SF) screening tool was used to screen the risk of malnutrition. The MNA is divided into screening and global assessment, and both parts were used in the research (15). Recent unintentional weight loss and reduced food intake were self-reported.

For stages 1 and 2 of the GLIM, which involve diagnosing and classifying the severity of malnutrition, all the GLIM criteria were applied (4). For step 1, phenotypic criteria, the BMI was categorized into moderately low, if BMI <20 kg/m^2^ (<22 if over 70 years old), and severely low, if BMI <18.5 kg /m^2^ (<20 if over 70 years old). For weight loss in the last 6 months, a cutoff point of 10% was used. Low body muscle mass, as measured by BIA, was defined as a FFMI (FFM (kg)/height (m^2^)) <15 kg/m^2^ for women and < 17 kg/m^2^ for men, indicating moderate malnutrition, according to the FFM cutoff values (12, 16). No additional FFMI cutoffs were used to define severe malnutrition as this is not specified in the original GLIM publication (4).

For the etiological criteria assessing participants’ disease/inflammation burden, the following were considered: acute disease, acute chronic disease, infection, or injury, all of which are generally associated with inflammatory activity. This criterion was supported by C-reactive protein (CRP) assessment (> 5 mg/L, when the contribution of inflammatory components was uncertain) (17, 18). The other etiological criteria were obtained through interviews, and malabsorptive intestinal conditions were collected from the patients’ medical records. For step 2 of the GLIM process, malnutrition severity was categorized as moderate by BMI and/or weight loss of 5 to 10% and/or reduced FFMI (19), using cutoff points CC < 33 cm for women and < 34 cm for men [21]. Participants were classified as severely malnourished by BMI and/or weight loss >10% [4] and/or CC < 31 cm for women and < 32 cm for men (20).

### 2.5 Mini nutritional assessment (MNA)

To assess the risk of malnutrition and the presence of malnutrition, MNA, (15) was applied. Isolated screening was used in the initial part of the GLIM tool. It consists of six items pertaining to food intake, involuntary weight loss, mobility, acute disease or psychological stress, neuropsychological problems, and BMI. A score of 12 or greater indicates that the individual was well nourished and needs no further intervention. A score of 8 to 11 indicates that the person was at risk of malnutrition, and a score of 7 or less indicates that the individual was malnourished. The complete MNA-FF was also used for nutritional diagnosis, as it is considered the gold standard for older adults. The first part of MNA-SF (short form) comprises six questions. The second part consists of 12 questions with a maximum score of 16 points. At the end, the scores were added, and patients were classified as having normal nutritional status (score > 23.5), and at risk of malnutrition (score 17–23.5) or malnourished (score < 17).

### 2.6 Sarcopenia

Sarcopenia diagnosis was conducted according to the algorithm suggested in the European consensus on sarcopenia. Individuals at risk of sarcopenia were assessed using the SARC-F muscular strength assessment via dynamometry. If the measured strength was reduced (< 27 KgF for men and < 16 KgF for women), the test result was considered positive for probable sarcopenia, indicating the need for further confirmation of low muscle quantity and quality (21). This was carried out using BIA appendicular skeletal muscle mass (ASMM) values, being considered reduced if <20 kg for men and < 15 kg for women, resulting in a positive diagnosis for sarcopenia (12).

### 2.7 Frailty

Frailty syndrome was screened using the self-referred frailty instrument developed by Nunes et al. (2015) (22). Participants were classified as frail if they exhibited three or more of the five components, pre-frail with one or two components present, and not frail if none of the components were met.

### 2.8 Statistical analysis

For statistical analysis, the Statistical Package for the Social Sciences (SPSS) version 21.0 (SPSS Inc., Chicago, IL, USA) was used. The Kolmogorov–Smirnov test was used for continuous variables. Student’s *t*-test was then applied to continuous data with normal distribution. For categorical variables, the chi-square test was performed. When expected cell values were less than 5, Fisher’s exact test was considered. Pearson’s correlation was used for normal distribution.

The statistical analyses recommended by Van Der Schueren were used to validate the GLIM criteria (23), using individuals with any combination of phenotypic and etiological criteria. Sensitivity, specificity, positive predictive value (PPV), and negative predictive value (NPV) they were calculated using the association of the MNA-SF screening instrument with the other GLIM criteria versus MNA-FF. Sensitivity and specificity of >80% were interpreted as acceptable for malnutrition diagnosis, and agreement between instruments was calculated using Cohen’s kappa. The level of agreement was interpreted as almost perfect if Cohen’s kappa (k) was >0.91, strong if k = 0.81–0.90, moderate if k = 0.60–0.80, weak if k = 0.40–0.59, and minimum if k < 0.40. Binomial logistic regression analysis was performed to assess relationships between sarcopenia, frailty, and malnourished by MNA-FF, GLIM ANTHRO, and GLIM BIA. Adjustments were made in adjustment for sex and age. The results were expressed with odds ratios (ORs) and 95% confidence intervals (95%CIs). The results that presented a significance level lower than 0.05 were considered significant.

## 3 Results

**Figure 1:**
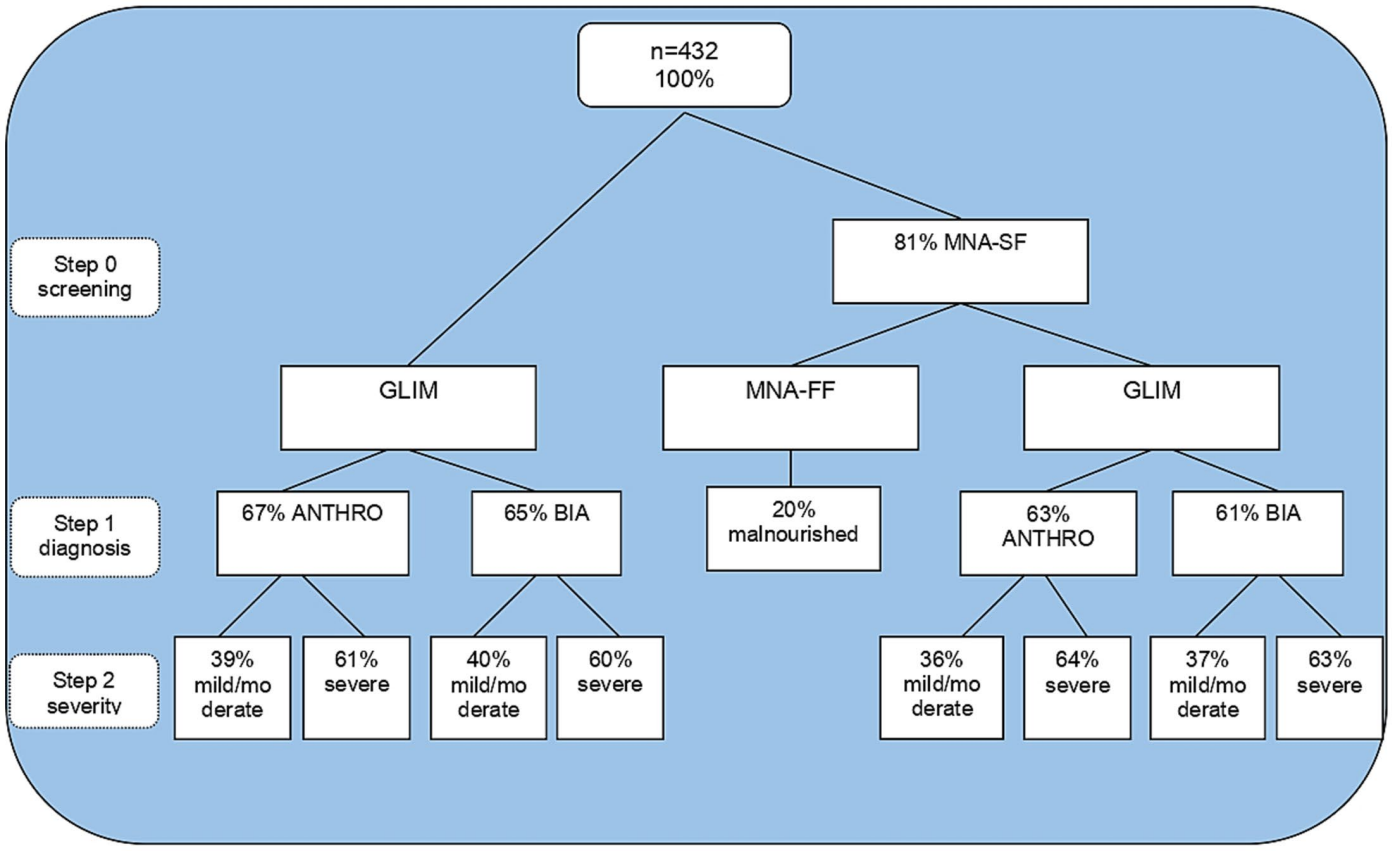
Steps of malnutrition diagnosis by GLIM and MNA. GLIM, global leadership initiative on malnutrition; MNA-SF, mini nutritional assessment short form; ANTHRO, anthropometry; BIA: bioelectrical impedance analysis.

A total of 432 individuals aged 60 or over were eligible, and their sociodemographic, clinical, and anthropometric characteristics are described in Table 1. The results of the total GLIM process are reported in Figure 1. Using MNA-SF as a screening tool in step 0, 81% were observed to be at risk of malnutrition. Depending on the methodology in step 1, it was observed that 36–37% of participants were categorized as mildly/moderately malnourished, and 63–64% of participants were categorized as severely malnourished. Meanwhile, MNA-FF found only 20% malnourished. When applying GLIM independent of screening, the proportion of malnourished adults over 60 years old was higher for step 1 and lower than GLIM with screening only for severe malnutrition.

**Table 1:** Characterization and association of sociodemographic, clinical, and anthropometric variables with malnutrition using GLIM ANTHRO.

	Total subjects *n* = 432	*p*-value (Total subjects)	Men *n* = 200	Women *n* = 232
**Age, mean in years** ± **SD**	71.14 ± 7.99	0.540	69.83 ± 7.46	72.27 ± 8.27
**Sex *n* (%)**	432 (100)	0.853	200 (46.3)	232 (53.7)
Admission diagnosis, *n* (%)				
Respiratory diseases	103 (23.8)		53 (26.5)	50 (21.6)
Gastrointestinal diseases	93 (21.5)		42 (21)	51 (22)
Neoplasms	72 (16.7)		35 (17.5)	37 (15.9)
Others	61 (14.1)	0.005*	29 (14.5)	28 (12.1)
Neurological disease	39 (9.0)		17 (8.5)	22 (9.5)
Infection	17 (3.9)		5 (2.5)	12 (5.2)
Hematological disease	12 (2.8)		6 (3.0)	6 (2.6)
Orthopedic/bone disease	11(2.5)		3 (1.5)	8 (3.4)
Kidney diseases	10(2.3)		1 (0.5)	9 (3.9)
Cardiovascular disease	9 (2.1)		5 (2.5)	4 (1.7)
Endocrine disease	5 (1.2)		3 (1.5)	2 (0.9)
Dysphagia, *n* (%)				
Does not present	372 (86.1)		180 (90)	192 (82.8)
Solid	25 (5.8)	0.087	7 (3.5)	18 (7.8)
Liquid	9 (2.1)		4 (2.0)	5 (2.2)
Solid and liquid	26 (6.0)		9 (4.5)	17 (7.3)
Anthropometry, mean ± SD				
Weight, kg	62.83 ± 15.47	0.884	66.12 ± 15.31	59.99 ± 15.07
Height, m	1.58 ± 0.09	0.173	1.65 ± 0.06	1.52 ± 0.07
BMI, kg/m^2^	24.99 ± 5.50	0.392	24.28 ± 4.92	25.61 ± 5.90
AC, cm	27.76 ± 4.91	0.524	27.40 ± 4.59	28.07 ± 5.16
CC, cm	33.01 ± 5.17	0.669	33.54 ± 5.95	32.59 ± 4.35
HGS, kgf	19.85 ± 9.39	0.210	26.36 ± 8.60	14.24 ± 5.71
Body composition, mean ± SD				
FM, kg, BIA	20.70 ± 13.64	0.495	17.03 ± 10.93	23.85 ± 14.91
SMM, kg, BIA	19.10 ± 6.72	0.762	23.52 ± 5.06	15.30 ± 5.54
FFM, kg, BIA	42.19 ± 9.55	0.469	49.04 ± 8.36	36.28 ± 5.86
FFMI, kg/m^2^	16.62 ± 2.61	0.901	17.91 ± 2.63	15.50 ± 2.01

Classification of GLIM ANTHRO criteria after MNA-SF screening. GLIM, global leadership initiative on malnutrition; ANTHRO, anthropometry; SD, standard deviation; BMI, body mass index; AC, arm circumference; CC, calf circumference; HGS, hand grip strength; BIA, electrical bioimpedance analysis; FM, fat mass; SMM, skeletal muscle mass; FFM, fat-free mass; FFMI, fat-free mass index. *P-value, statistically significant (*p* < 0.05). Pearson’s chi-square test, Fisher’s exact test, or Student’s *t*-test.

Data relating to the GLIM criterion in steps 1 and 2 (without applying step 0) demonstrated that by anthropometry, 67% of the assessed population was diagnosed as malnourished. Of these, 39% were moderately malnourished, and 61% were severely malnourished.

Using bioelectrical impedance, the occurrence of 65% of malnutrition was found. Of these, 40% were moderately malnourished and 60% severely malnourished. Weight loss and reduced food intake were the most frequent combination that led to malnutrition diagnosis, followed by weight loss and inflammation for both GLIM methodologies. The combination of low BMI and inflammation was also the least common for both methods according to Table 2.

**Table 2:** Results of GLIM steps 1 and 2 without nutritional screening.

Process step GLIM	Criterion	GLIM ANTHRO		GLIM BIA	
		*n*	%	*n*	%
**Step 1: Diagnosis**	**Combinations of malnutrition**	**290**	**67**	**279**	**65**
	Weight loss >5% in 6 months/>10% in >6 months and reduced intake/assimilation	206	71	206	74
	Weight loss >5% in 6 months/>10% in >6 months and inflammation	175	60	175	63
	BMI < 20/22 kg/m^2^ and reduced food intake/assimilation	83	29	83	30
	BMI < 20/22 kg/m^2^ and inflammation	75	26	75	27
	Low FFMI and reduced dietary intake/assimilation			112	40
	Low FFMI and inflammation			110	39
	Low CC and reduced dietary intake/assimilation	159	55		
	Low CC and inflammation	154	53		
**Step 2: Severity**	**Mild/moderate malnutrition, total**	**114**	**39**	**112**	**40**
	BMI 20–22 kg/m^2^	6	5	6	5
	Weight loss 5–10% in 6 months/10–20% > 6 months	75	66	75	67
	FFMI reduction			52	46
	CC reduction	52	46		
	**Severe malnutrition**	**176**	**61**	**167**	**60**
	BMI < 18.5 kg/m^2^ in <70 years/<20 in ≥70 years	57	32	57	34
	Weight loss >10% in 6 months/> 20% in >6 months	123	70	123	74
	Severe CC reduction	126	71		

*N = 432. GLIM, global leadership initiative on malnutrition. BMI, body mass index. CC, calf circumference. FFMI, fat-free mass index; BIA, bioelectrical impedance analysis. The values in bold refer to the total n and percentage of each stage evaluated.*

The agreement between the GLIM criteria, both with and without screening, and MNA-FF is presented in Table 3. The GLIM process identified more individuals than the MNA-FF for both GLIM ANTHRO and GLIM BIA. When considering the MNA-FF as a reference method, GLIM sensitivity reached acceptable levels, with and without screening, regardless of the methodology. However, specificity was significantly reduced, though it increased slightly when screening tools were employed compared to GLIM without screening. The agreement between the MNA-FF and GLIM ANTHRO criteria with screening was minimal (kappa −0.11) as well as using the GLIM BIA with screening (kappa −0.11), and those without screening obtained a kappa of −0.10 for GLIM ANTHRO and kappa of −0.10 for GLIM BIA. NPV was acceptable for both GLIM methodologies with and without screening, increasing with the use of screening, indicating that GLIM had a high probability of assessing individuals as not malnourished and that they actually did not have a malnutrition diagnosis, unlike PPV.

**Table 3:** Agreement in malnutrition diagnosis by MNA and GLIM ANTHRO/BIA.

	GLIM ANTHRO						
			Malnutrition			Specific agreement	
Screening step 0 (*n* = 432)	MNA (*n*)	GLIM ANTHRO (*n*)	Sensitivity (%)	Specificity (%)	Kappa	PPV %	NPV %
Without screening	87	290	92	39.1	−0.108	27	95
MNA-SF	87	272	92	44.3	−0.117	29	96

	**GLIM BIA**						
			Malnutrition			Specific agreement	
Screening step 0 (*n* = 432)	MNA (*n*)	GLIM BIA (*n*)	Sensitivity (%)	Specificity (%)	Kappa	PPV %	NPV %
Without screening	87	279	90.8	42.0	−0.109	28	91
MNA-SF	87	265	90.8	46.1	−0.116	30	95

GLIM, global leadership initiative on malnutrition; ANTHRO, anthropometry; BIA: bioelectrical impedance analysis; MNA-SF, mini nutritional assessment short form; PPV, positive predictive value, NPV, negative predictive value.

Table 4 presents the association, determined through logistic regression, between malnutrition assessed using the GLIM tools (both methods) and the MNA-FF, as well as the health conditions of the individuals at the time of hospital admission. It was possible to observe that both in the unadjusted analysis and in the analysis adjusted for sex and age, individuals with malnutrition assessed by the MNA-FF and GLIM ANTHRO had a greater chance of sarcopenia and frailty, which differed from the assessment using the GLIM BIA, which showed an increased risk only for frailty.

**Table 4:** Logistic regression analysis between GLIM and MNA-FF tools and health conditions.

Health condition status	Malnourished (MNA-FF)		Malnourished GLIM ANTHRO			Malnourished GLIM BIA	
	Unadjusted		Unadjusted			Unadjusted	
	OR (95% CI)	*p*-value	OR (95% CI)	*p*-value		OR (95% CI)	*p*-value
**Sarcopenia**	3.085 (1.849–5.147)	**<0.001**	1.666 (1.055–2.630)	**0.028**		1.410 (0.901–2.209)	0.133
**frailty**	15.995 (2.161–118.361)	**0.007**	2.213 (1.318–3.715)	**0.003**		2.454 (1.458–4.129)	**0.001**
**Health condition status**	**Malnourished (MNA-FF)**		**Malnourished GLIM ANTHRO**			**Malnourished GLIM BIA**	
	Adjustment		Adjustment			Adjustment	
	OR (95% CI)	*p*-value	OR (95% CI)	*p*-value		OR (95% CI)	*p*-value
**Sarcopenia**	3.097 (1.855–5.173)	**<0.001**	1.682 (1.063–2.662)	**0.026**		1.417 (0,902–2.227)	0.130
**Frailty**	15.976 (2.158–118.251)	**0.007**	2.223 (1.322–3.736)	**0.003**		2.255 (1.455–4.141)	**0.001**

GLIM, global leadership initiative on malnutrition; MNA-FF, mini nutritional assessment full form; ANTHRO, anthropometry; BIA, bioelectrical impedance analysis. *Statistically significant *p*-value (*p* < 0.05); OR, odds ratio. 95% CI, 95% confidence interval. Adjustment, sex and age. Values in bold refer to statistically significant values.

## 4 Discussion

This study shows that the frequency of malnutrition diagnosed by the GLIM method was higher than the MNA-FF and that this tool does not identify malnutrition severity. GLIM anthropometry and GLIM bioimpedance had very similar implications, suggesting that, when using GLIM references to assess muscle mass, regardless of the method, the findings are analogous and the values agree with the prevalence of malnutrition in the studied population (24). The disagreement between the MNA-FF and GLIM methods was generally very high, being greater when the MNA-SF tool was not used.

### 4.1 Effect of reduced MNA-SF tool on GLIM results

When comparing our results with the literature, it was possible to verify that the percentage of malnourished individuals using the MNA-SF and all GLIM criteria was higher than that found by other authors (25, 26). Although all studies use the MNA-SF as a screening step, divergences in the prevalence of malnutrition are understandable due to population differences, the number of GLIM criteria adopted, and the measurement of muscle mass, which should follow the proposed guidelines (19).

Specificity, PPV, and NPV increased when comparing the GLIM and MNA-FF when screening was applied. The MNA-FF identified a relatively high proportion of individuals “at risk of malnutrition,” confirming its high sensitivity (25). In this study, the number of malnourished diagnoses without screening was only 4% higher than those who used this step. This suggests that the use of screening resulted in a reduced number of malnourished individuals who could not be identified, another study found an even higher number of malnourished people who were not identified using screening (27). Other authors also found that, even though they were classified as malnourished by the GLIM, some older adults did not present nutritional risk according to the MNA-SF (28, 29).

GLIM sensitivity was maintained without screening, demonstrating that GLIM can verify malnourished older adults regardless of screening. This can be supported by the fact that none of the existing screening tools are able to detect low muscle mass (30). In a study using the GLIM and MNA-FF, a sensitivity of 76% was observed (25). A similar sensitivity of 75% was also verified when analyzing older adults in the community with the MNA (31). Therefore, when comparing the tools, given the sensitivity and specificity of screening, as well as better identification of malnutrition severity, it is suggested that the screening tool be used in GLIM.

The kappa value showed insignificant variation when comparing the MNA-FF method to the GLIM with and without screening. This agreement could be explained since MNA-FF does not assess the reduction in FFM, inflammation, or weight loss prior to 3 months, in the same way as the GLIM, which goes much less in-depth into nutritional assessment, such as when a body composition index is measured using the GLIM. In addition, the MNA-SF uses a different BMI scaling system and a lower cutoff point. Thus, although the MNA is suitable for risk and malnutrition, the GLIM may be more efficient for detecting hidden malnutrition, including sarcopenic obesity.

### 4.2 Agreement between GLIM and MNA

It was observed that one study compared the GLIM with the MNA-FF (28), and only a few had applied the MNA-SF followed by the GLIM to diagnose malnutrition in hospitalized older adults (25, 26, 28, 29, 32). It was found that the GLIM without screening identified more than three times the number of malnourished individuals as the MNA-FF. Other authors, using FFMI, observed a disparity in malnutrition rates: 36% identified as malnourished by GLIM compared to 15% by PG SGA (33). Similar values were found without using FFMI (34), in contrast to other studies that reported lower malnutrition prevalence with GLIM (27) and Ref. (35).

GLIM sensitivity was greater than 90% in analysis without screening, being higher than the results presented in the literature (33, 34, 36). The PPV without screening was close to some studies, at 34% (36) and 29% (33), but lower when compared to PPV of 83% (36). The kappa value without the screening step indicated a very low agreement between the GLIM and MNA-FF, being lower than the kappa of 0.32 (34) and 0.45 (35).

Discrepancies in comparisons can be partially explained by differences in the criteria used in each tool. Many studies do not use all the GLIM criteria and focus on populations from different regions and socioeconomic conditions. Additionally, tools, such as the MNA-FF do not use the same diverse criteria for identifying malnutrition as GLIM does.

### 4.3 Comparison between MNA-FF and GLIM and health conditions

Malnutrition identified by GLIM ANTHRO and MNA-FF was associated with sarcopenia, an association already reported by a systematic review with meta-analysis (37), confirming the premise that ignoring malnutrition can lead to sarcopenia and that the concomitance of these conditions is defined as malnutrition-sarcopenia syndrome (38). Older adults admitted with the syndrome have twice the risk of death as malnourished or sarcopenic adults alone (39). However, some individuals with sarcopenia, in this and another study, did not show malnutrition at screening, which further emphasizes the need to make a complete diagnosis for both malnutrition and sarcopenia (40).

Regarding frailty, an association with malnutrition was found by GLIM and MNA-FF, with a much higher risk when using the MNA-FF, as observed in hospitalized frail older adults (26) and adults over 60 years old (41). In homes of older adults, the coexistence of three conditions was also observed, such as malnutrition, frailty, and physical dysfunction (42).

MNA-FF and GLIM ANTHRO had a better association with health conditions than GLIM BIA, possibly because BIA is more affected by clinical conditions in hospitalized patients, which can alter tissue physiology, than muscle assessment by calf circumference. BIA estimates total body water and other body compartments using predictive equations that assume constant tissue hydration in individuals (43). However, this assumption may not always occur during hospitalization. In addition, anthropometry and bioimpedance methods express different aspects and levels of nutritional deficiency (44). However, it was also possible to observe that MNA-FF was a better predictor of frailty and sarcopenia in individuals than GLIM.

## 5 Conclusion

It was possible to verify that the MNA-FF and GLIM result in low agreement as they use divergent conceptual frameworks to understand malnutrition, which subsequently leads to different prevalences of malnutrition. However, although the GLIM ANTHRO obtained a greater number of malnutrition diagnoses and showed a good association with frailty and sarcopenia, more studies are needed to support its use in the diagnosis of malnutrition in hospitalized populations over the age of 60, since the MNA-FF was able to predict sarcopenia and frailty with a higher hazard ratio.

## Data Availability

The original contributions presented in the study are included in the article/Supplementary material, further inquiries can be directed to the corresponding authors.
