# Supplementary figures and images for: The ability of GLIM and MNA-FF to diagnose malnutrition and predict sarcopenia and frailty in hospitalized adults over 60 years of age
Source: Front Nutr. 2024 Nov 8;11:1456091. doi: 10.3389/fnut.2024.1456091 (PMC11583805; doi:10.3389/fnut.2024.1456091)

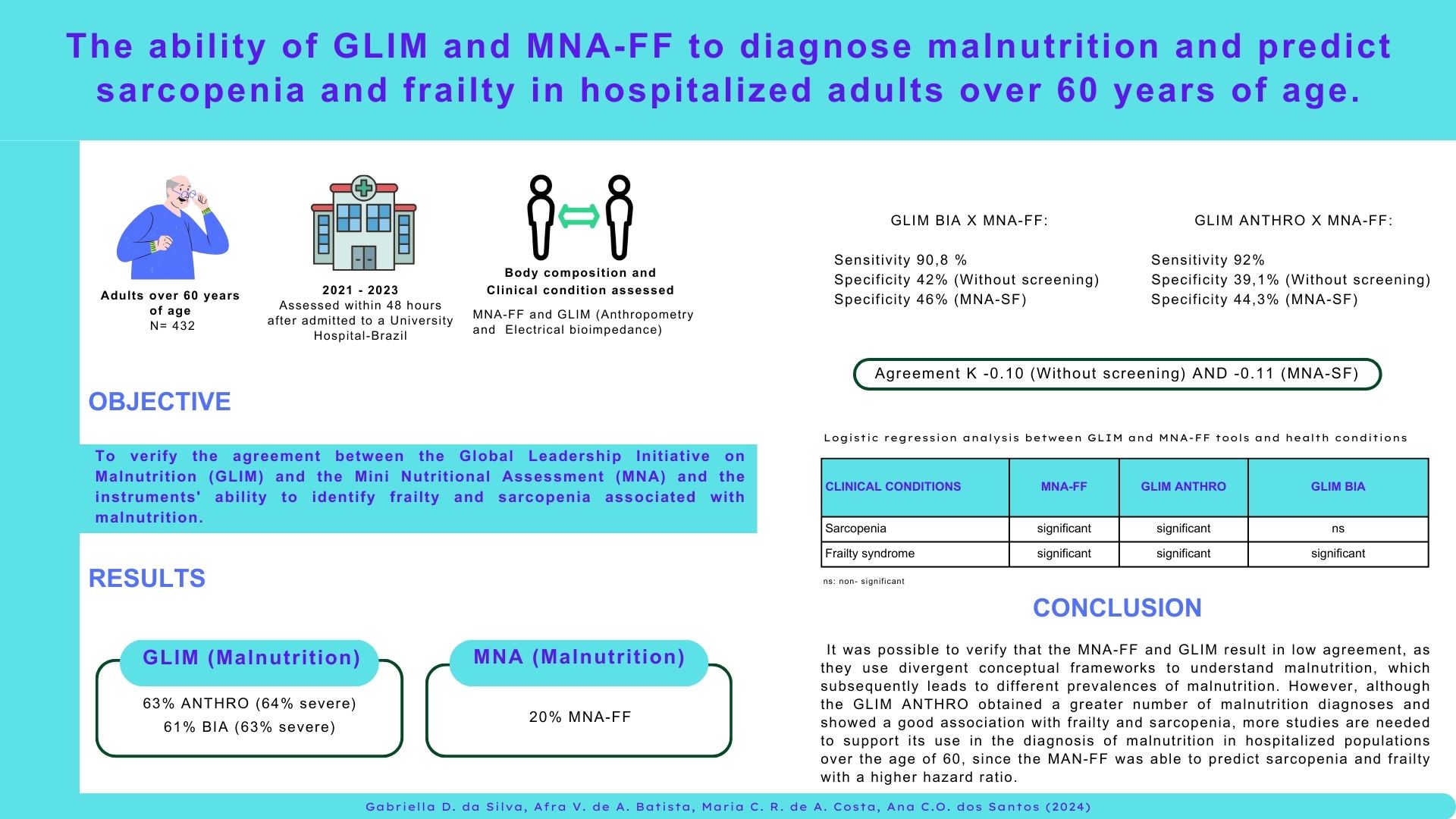

Supplement: Supplementary file 1 [file Image_1.jpg]
